# Systemic Treatment of Advanced Chordoma With Molecular Targeted Therapies: A Large Multicentre European Retrospective Case Series

**DOI:** 10.1002/cnr2.70596

**Published:** 2026-06-11

**Authors:** Astrid Lipplaa, Nadia Hindi, Pawel Teterycz, Robin J. Young, Ioannis Boukonavis, Josefina Cruz, Javier Martinez‐Trufero, Frank Speetjens, Wilco Peul, Robert J. P. van der Wal, Hans Gelderblom

**Affiliations:** ^1^ Department of Medical Oncology Leiden University Medical Center Leiden the Netherlands; ^2^ Department of Oncology Hospital Universitario Virgen del Rocío Seville Spain; ^3^ Department of Soft Tissue/Bone Sarcoma and Melanoma Maria Sklodowska‐Curie Institute ‐ Oncology Center Warsaw Poland; ^4^ Department of Oncology Weston Park Hospital Sheffield UK; ^5^ Department of Oncology Aristotle University of Thessaloniki Thessaloniki Greece; ^6^ Department of Oncology Hospital Universitario de Canarias San Cristóbal de La Laguna Spain; ^7^ Department of Oncology Hospital Universitario Miguel Servet Zaragoza Spain; ^8^ Department of Neurosurgery Leiden University Medical Center Leiden the Netherlands; ^9^ Department of Orthopaedic Surgery Leiden University Medical Center Leiden the Netherlands

**Keywords:** chordoma, molecular targeted therapy, systemic therapy, tyrosine kinase inhibitors

## Abstract

**Background:**

This retrospective series reports outcomes and prognostic factors for advanced chordoma (AC) treatment with molecular targeted therapies (MTTs) in different treatment lines.

**Methods:**

This is a retrospective series of 57 patients with AC treated between 2004 and 2023 at one of seven participating sarcoma centres. Demographics, previous treatment, treatment details and outcomes were recorded.

**Results:**

57 patients were treated with 7 different MTTs. Treatment was received in first (*n* = 57), second (*n* = 16), third (*n* = 5) or fourth (*n* = 1) line. The most frequently administered agent in first line was imatinib (84.2%) and in second line imatinib + sirolimus (35.7%). Overall median progression free survival (PFS) and overall survival (OS) in first‐line treatment was 6.5 (95% CI 4.0–9.0) and 29.5 months (95% CI 24.0–40.4) and in second‐line 10.0 (95% CI 4.0–22.0) and 37.2 months (95% CI 9.4–45.9). Partial response according to RECIST 1.1 was seen in 5/79 treatments (6.3%). Dose reductions and interruptions were reported in 19.0% and 27.8% of treatments.

**Conclusions:**

PFS and response rates with these MTTs were in line with previous phase II trials and retrospective series. Although the efficacy does not meet the European Society of Medical Oncology (ESMO) Magnitude of Clinical Benefit Scale (MCBS) criteria for single arm studies in orphan diseases, MTTs are frequently used off‐label due to the high unmet need and lack of other systemic treatment options. The toxicity profile and limited efficacy rate should be taken into account when counselling patients. Further research is needed to explore other systemic treatment options including (combinations with) immunotherapy.

**Abbreviations**
AC: advanced chordoma
ALK: anaplastic lymphoma kinase
A.o.: among others
ASPS: alveolar soft part sarcoma
CI: confidence interval
CS: chondrosarcoma
CTLA‐4: cytotoxic lymphocyte associated protein 4
ECOG: eastern cooperative oncology group
EGFR: epidermal growth factor receptor
ESMO: European society of medical oncology
HR: hazard ratio
IGF‐1R: insulin‐like growth factor‐1 receptor
IL2: interleukin 2
IQR: interquartile range
LAG3: lymphocyte‐activation gene 3
MCBS: magnitude of clinical benefit scale
mTOR: mechanistic target of rapamycin
MTT: molecular target therapies
ORR: objective response rate
OS: overall survival
PD: progressive disease
PDGFR: platelet derived growth factor‐receptor
PD(L)‐1: programmed cell death (ligand) protein 1
PFS: progression free survival
PR: partial response
RECIST: response evaluation criteria in solid tumours
SFT: solitary fibrous tumour
TKI: tyrosine kinase inhibitor
VEGFR: vascular endothelial growth factor receptor

## 1 Background

Chordomas are rare bone tumours affecting the axial skeleton and skull base that show notochordal differentiation [1]. Although these tumours are typically slow‐growing, they exhibit locally aggressive behaviour. While distant metastases can occur, locoregional recurrences are more common and are associated with considerable morbidity and mortality. Due to its low incidence of 1:1.000.000 the treatment of chordoma is highly centralised [2]. Chordomas are categorised as either conventional chordomas (including chondroid types), poorly differentiated or dedifferentiated chordomas. Loss of SMARCB1 (INI1) expression is characteristic of poorly differentiated chordomas [3]. Finally, dedifferentiated chordomas are characterised by rapid growth, occurrence at a younger age and a higher likelihood of metastases [1].

The mainstay of treatment is wide surgical resection, which is often challenging depending on tumour extension and anatomical site [4]. The high rate of local disease relapse is strongly related to the resection margins at primary surgery [5, 6]. Radiotherapy is recommended as adjuvant therapy to surgery or as definitive treatment in case surgical resection is not feasible or not desirable by the patient due to the morbidity of the surgical resection [4]. An adequate dose of above 70 Gy is needed due to the radioresistant nature of these tumours and the use of particle beam therapy (proton and/or carbon beam) leads to better local control and less damage to surrounding structures [7]. Chordomas are considered advanced when a localised recurrence is multifocal, metastatic or not amenable to local therapies such as surgery or high‐dose radiotherapy [4].

Cytotoxic chemotherapies are known to have a very limited efficacy and are not currently recommended in any line of treatment for conventional chordoma [4, 8]. There has been great interest in the potential role of molecular targeted therapies (MTTs) in the treatment of advanced chordoma (AC). However, to date, all data on MTT is derived from small phase II studies or case series. To date, most evidence is available for platelet derived growth factor receptor (PDGFR) inhibitor imatinib, which has shown positive results in a phase II study in (AC) [9].

This retrospective series aims to give more insight into outcomes and prognostic factors in AC patients who have been treated with MTTs as first‐line or later line treatment, to reinforce findings from previous case series and small phase 2 trials, using real‐world data and to help guide clinicians in making optimal treatment choices for patients with AC.

## 2 Patients and Methods

### 2.1 Patients

This large multicentre European retrospective series includes 57 adult patients with histologically proven progressive, locally advanced or metastatic chordoma treated with MTT between 2004 and 2023. Patients were recruited across multiple centres, with each patient treated at a single participating sarcoma centre (Leiden University Medical Center, Leiden, The Netherlands; Weston Park Hospital, Sheffield, United Kingdom; Maria Sklodowska‐Curie Institute, Warsaw, Poland; Hospital Universitario Virgen del Rocío, Seville, Spain; Hospital Universitario Miguel Servet, Zaragoza, Spain; Hospital Universitario de Canarias, Tenerife, Spain; Aristotle University of Thessaloniki, Thessaloniki, Greece). Subjects with AC, who were no longer amenable to local therapies such as surgery or radiotherapy, were included. Systemic treatment options included MTT; cytotoxic chemotherapy was excluded. The following patient data were collected retrospectively: age, gender, year of diagnosis, Eastern Cooperative Oncology Group (ECOG) performance status, histologic subtype, tumour location, tumour extension, location of metastases, previous treatments, resection status of previous surgery, type of MTT, dose of MTT, age at treatment start, progression free survival (PFS), overall survival (OS), best response, symptomatic improvement, major toxicity (grade ≥ 3), dose reductions, dose interruptions and date of last contact or death. The term ‘treatment line’ refers to the different TKI treatments given sequentially, progress to a new line was initiated upon progression of disease or poor treatment tolerance. Primary endpoints were objective response, PFS and OS. These were assessed by response evaluation criteria in solid tumours RECIST version 1.1 [10] or on clinical grounds when no RECIST assessment was available. Secondary endpoints were toxicity and dose adaptations.

### 2.2 Statistical Analysis

Overall survival (OS) and PFS were calculated using the Kaplan–Meier method and compared with the log‐rank test. Univariate analyses were performed using Cox regression analysis. Different treatment lines within the same subject were analysed separately for OS and PFS.

### 2.3 Ethics

The study was approved by the local Ethics Committee at the Leiden University Medical Center in Leiden, The Netherlands (approval reference number 180319EKA). Ethical approval from other participating centres and written consent from individual patients was not required in compliance with Dutch and European laws governing non‐interventional studies (https://english.ccmo.nl/investigators/additional‐requirements‐for‐certain‐types‐of‐research/non‐wmo‐research/file‐research).

## 3 Results

A total of 57 AC patients were treated with MTTs in the given time period and analysed in this series.

### 3.1 Patient Characteristics

The main baseline characteristics of the 57 included patients are summarised in Table 1. The majority of patients were male (61.4%) and the median age was 55 years (range 25–83).

**TABLE 1:** Baseline patient and clinical characteristics.

Characteristics		*n* (%)
Total number of patients		57
Gender	Male	35 (61.4)
	Female	22 (38.6)
Age (at treatment start)	Median, years [range]	55 [25–83]
ECOG performance status	0	10 (17.5)
	1	37 (64.9)
	2	7 (12.3)
	Unknown	3 (5.3)
Location primary tumour	Skull base	10 (17.5)
	Cervical spine	7 (12.3)
	Thoracic spine	2 (3.5)
	Lumbar spine	6 (10.5)
	Sacrum	32 (56.1)
Histological subtype	Conventional	50 (87.7)
	Chondroid	2 (3.5)
	Dedifferentiated	2 (3.5)
	Unknown	3 (5.3)
Tumour extension	Local	32 (56.1)
	Metastatic	25 (43.9)
Location metastases	Lung	12 (21.1)
	Bone	10 (17.5)
	Lymph node	6 (10.5)
	Subcutaneous	6 (10.5)
	Muscle	6 (10.5)
	Liver	5 (8.8)
	Other	4 (7.0)
Resection status surgery	R0	11 (19.3)
	R1	11 (19.3)
	R2	25 (43.9)
	Resection status unknown	6 (10.5)
	No prior surgery	4 (7.0)
Prior radiotherapy	Photon	31 (54.4)
	Hadron (proton/carbon ion)	12 (21.1)
	Photon + hadron	1 (1.8)
	Other	4 (7.0)
	No prior radiotherapy	9 (15.8)
Prior systemic therapy	Phase I	3 (5.3)
	Chemotherapy	2 (3.5)
	Unknown	1 (1.8)
	No prior systemic therapy	52 (91.2)

Abbreviation: ECOG: eastern cooperative oncology group.

Most patients (82%) were ECOG performance status 0 or 1 and had conventional chordoma (87%). As expected, most chordomas (56%) were sacral in origin with locally advanced disease (56%). Most patients had undergone previous surgery (93%) and radiotherapy (84%) and most (91%) had received no prior systemic treatment. See Table 1.

### 3.2 MTT and Response

**FIGURE 1:**
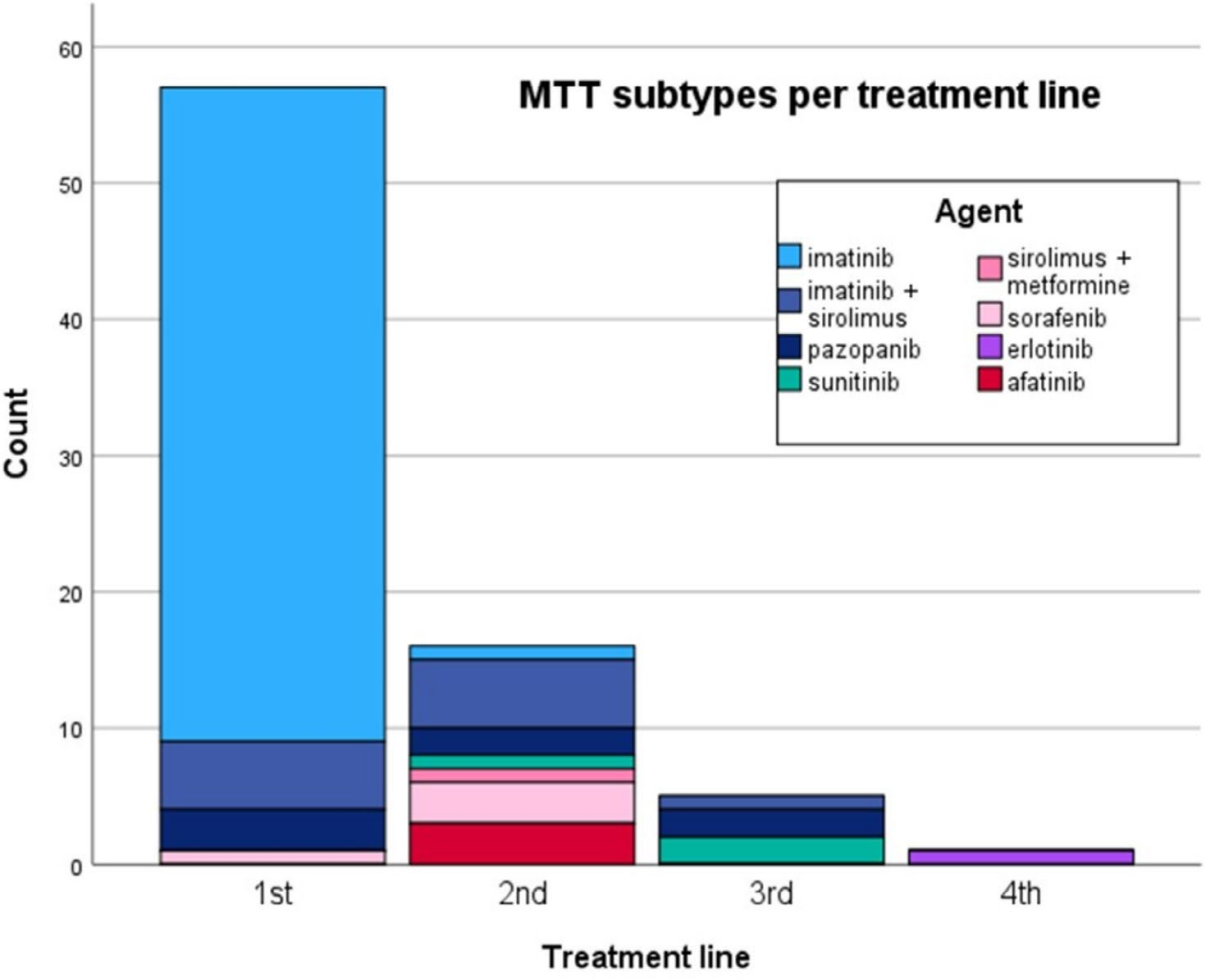
MTT subtypes per treatment line.

Of the 57 patients, 41 were treated with a single line of MTT, 11 were treated with two sequential lines of MTT, four received three lines and one subject four lines; 79 treatment lines were prescribed in total in 57 individual patients. The most frequently prescribed MTT in first line was the PDGFR inhibitor imatinib in 48/57 (84.2%) patients, followed by imatinib in combination with the mechanistic target of rapamycin (mTOR) inhibitor sirolimus in 5/57 (8.8%) and the multi‐kinase inhibitor pazopanib in 3/57 (5.3%). In second line, imatinib combined with sirolimus was most frequently prescribed, 5/14 (35.7%), followed by the multi‐kinase inhibitor sorafenib in 3/14 (21.4%) and the EGFR inhibitor afatinib in 3/16 (18.8%). All five patients treated with imatinib plus sirolimus in second line had received imatinib monotherapy in first line. Third line treatment consisted of pazopanib in 2/5 (40%), sunitinib in 2/5 (40%) and imatinib + sirolimus in 1/5 (20%). The only patient treated in fourth line received the epidermal growth factor receptor (EGFR) inhibitor erlotinib. Treatment with MTT started a median 5.0 years (range 2.7–7.7) after initial chordoma diagnosis. Median duration of treatment was 5.0 months (range 0.3–109); see also Table 2 and Figure 1.

**TABLE 2:** MTT characteristics per individual subject and individual treatment line (multiple treatment lines were administered within the same individual subject).

Characteristics per individual subject		*n* = 57
No lines of MTT per patient, no (%)	1	41 (71.9)
	2	11 (19.3)
	3	4 (7.0)
	4	1 (0.02)

Characteristics per individual treatment line		*n* = 79
Type MTT, no (%)	Imatinib	49 (62.0)
	Imatinib + sirolimus	11 (13.9)
	Pazopanib	7 (8.9)
	Sorafenib	4 (5.1)
	Sunitinib	3 (3.8)
	Afatinib	3 (3.8)
	Sirolimus + metformin	1 (1.3)
	Erlotinib	1 (1.3)
Interval between diagnosis and start MTT, median, years [IQR]	All	5.0 [2.7–7.7]
	1st line	4.6 [2.1–6.6]
	2nd line	5.8 [3.5–7.7]
	3rd line	8.0 [5.7–12.0]
	4th line	9.3 [–]

Abbreviations: CI: confidence interval, IQR: interquartile range, MTT: molecular targeted therapies.

Best radiological response was a partial response (PR) in 5/79 (6.3%) treatments, stable disease in 34/79 (43.0%), progressive disease in 31/79 (39.2%) and no RECIST response was available for 9/79 (11.4%) treatments. Most PRs were observed in first‐line treatment (4/5), 1/5 PR was in second line; see Table 3. The four patients with a PR on first‐line treatment had all received imatinib, the single patient with a PR on second line was treated with sunitinib (after first‐line treatment with imatinib resulting in a limited 4 months of stable disease). Overall response rate for imatinib treatment was 4/49 (8.2%) and for sunitinib 1/3 (33.3%). See Table 3.

**TABLE 3:** Results stratified by treatment line, MTT agent and tumour localization.

Results		
Progression free survival, median, months [95% CI]	All treatment lines (*n* = 79)	7.0 [5.0–10.0]
By treatment line	1st line treatment (*n* = 54)	6.5 [4.0–9.0]
	2nd line treatment (*n* = 16)	10.0 [4.0–22.0]
	3rd line treatment (*n* = 4)	4.5 [4.0–17.0]
	4th line treatment (*n* = 1)	4.0
	*(Data missing for 4 pts—3 imatinib, 1 pazopanib, see manuscript text)*	
By MTT agent	Imatinib (*n* = 46)	6.5 [4.0–9.0]
	Imatinib + sirolimus (*n* = 11)	10.0 [2.0–22.0]
	Pazopanib (*n* = 6)	14.5 [2.0–33.0]
	Sorafenib (*n* = 4)	5.0 [3.0–11.0]
	Sunitinib (*n* = 3)	5.0 [4.0–26.0]
	Afatinib (*n* = 3)	6.0 [3.0–20.0]
	Sirolimus + metformin (*n* = 1)	10.0 [–]
	Erlotinib (*n* = 1)	4.0 [–]
By primary tumour localization	Skull base (*n* = 12)	4.5 [2.0–10.0]
	Cervical spine (*n* = 8)	8.0 [2.0–13.0]
	Thoracic spine (*n* = 3)	7.0 [3.0–10.0]
	Lumbar spine (*n* = 12)	4.0 [4.0–22.0]
	Sacrum (*n* = 44)	7.5 [5.0–14.0]
Overall survival, median, months (95% CI)	All treatment lines (*n* = 79)	29.5 [23.8–39.1]
By treatment line	1st line treatment (*n* = 57)	29.5 [24.0–40.4)
	2nd line treatment (*n* = 16)	37.2 [9.4–45.9]
	3rd line treatment (*n* = 5)	20.8 [12.0–35.0]
	4th line treatment (*n* = 1)	8.0 [−]
By MTT agent	Imatinib (*n* = 49)	30.0 [24.0–40.4]
	Imatinib + sirolimus (*n* = 11)	39.1 [10.2–71.3]
	Pazopanib (*n* = 7)	29.5 [15.7–69.8]
	Sorafenib (*n* = 4)	7.2 [3.2–39.8]
	Sunitinib (*n* = 3)	16.5 [12.0–48.4]
	Afatinib (*n* = 3)	9.4 [6.0–53.0]
	Sirolimus + metformin (*n* = 1)	10.7 [−]
	Erlotinib (*n* = 1)	8.0 [−]
By primary tumour localization	Skull base (*n* = 12)	11.4 [8.4–23.8]
	Cervical spine (*n* = 8)	29.7 [5.2–72.0]
	Thoracic spine (*n* = 3)	19.1 [15.7–28.0]
	Lumbar spine (*n* = 12)	37.0 [12.0–54.6]
	Sacrum (*n* = 44)	38.7 [28.8–45.7]
Best radiological response, no (%)	Partial response	5 (6.3)
	Stable disease	34 (43.0)
	Progressive disease	31 (39.2)
	Unknown	9 (11.4)
Symptomatic improvement, no (%)	Yes	22 (27.8)
	No	44 (55.7)
	Unknown	13 (16.5)

Abbreviations: CI: confidence interval, MTT: molecular targeted therapies.

Patient reported symptomatic improvement was noted during 22/79 (27.8%) treatments, no improvement in 43/79 (54.4%) treatments and unknown in 13/79 (16.5%); see Table 3. In the five treatments resulting in a partial response, only two patients reported symptomatic improvement. See Table 3.

### 3.3 Progression Free Survival

**FIGURE 2:**
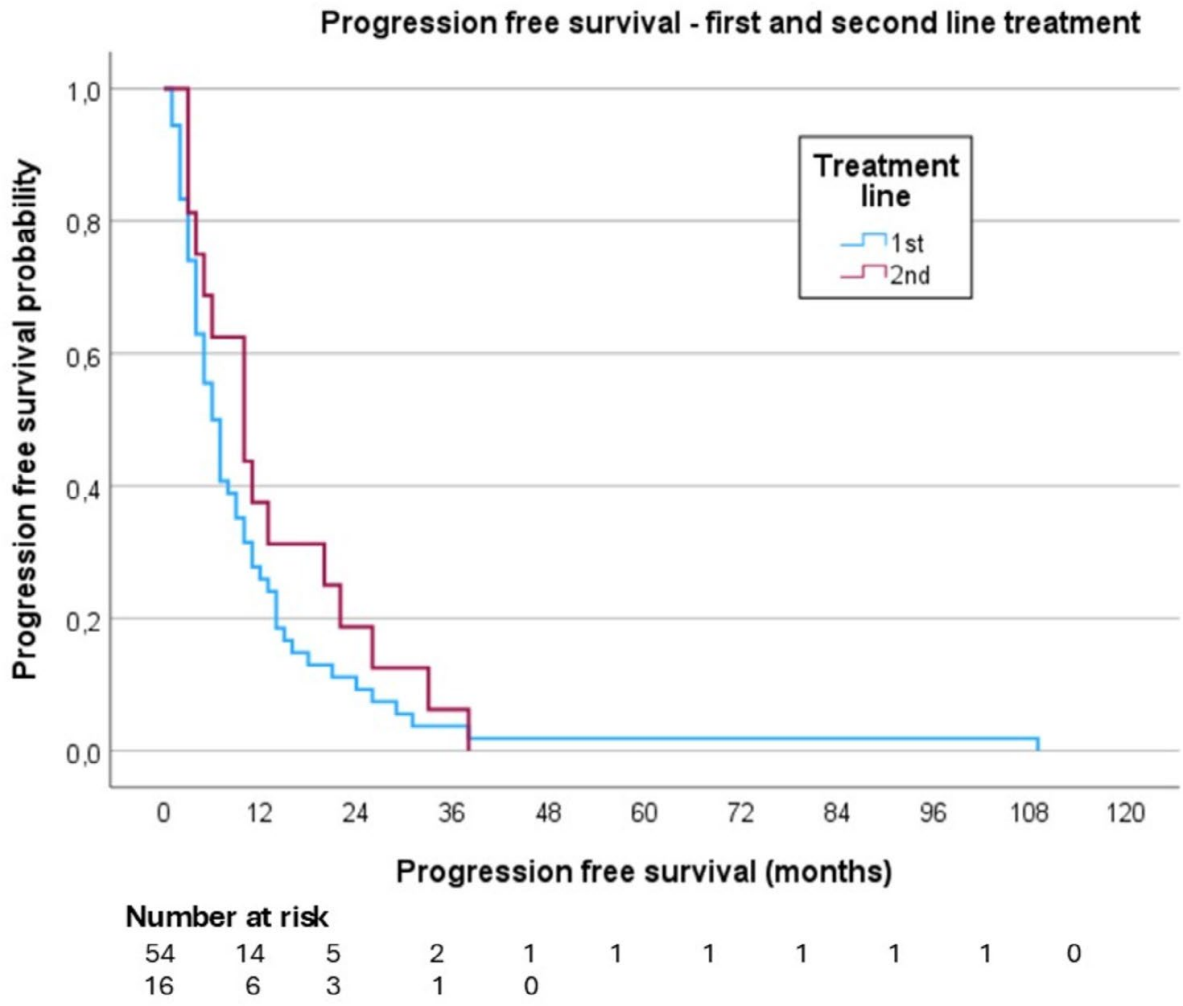
Progression‐free survival for first‐ and second‐line treatment. Median PFS: 1st line 6.5 months (95% CI 4.0–9.0); 2nd line 10.0 months (95% CI 4.0–22.0). PFS data unavailable for 3 first‐line treatments: 2 patients stopped due to toxicity without follow‐up; 1 subject was treated in neo‐adjuvant setting. CI: confidence interval; PFS: progression free survival.

Median PFS in first‐line treatments was 6.5 months (95% CI 4.0–9.0). In second line this was 10.0 months (95% CI 4.0–22.0), third line 4.5 months (4.0–17.0) and the fourth line treatment showed a PFS of 4.0 months; see Table 3 and Figure 2. In the five treatments with a partial response, median PFS was 7 months (range 4–26) and median OS 48.0 months (range 12.9–57.2).

Median PFS for imatinib treatments was 6.5 months (95% CI 4.0–9.0), imatinib + sirolimus 10.0 months (95% CI 2.0–22.0), pazopanib 14.5 months (95% CI 2.0–33.0), sunitinib 5.0 months (95% CI 4.0–26.0), sorafenib 5.0 months (95% CI 3.0–11.0) and afatinib 6.0 months (95% CI 3.0–20.0); see Table 3.

Median PFS on imatinib + sirolimus in first line was 10.0 months (95% CI 2.0–21.0), whereas median PFS in second line was 13.0 months (95% CI 4.0–38.0).

PFS data was unavailable for three patients in first‐line treatment (2 patients stopped due to toxicity without follow‐up, 1 subject stopped MTT and had surgery) and one patient in third‐line treatment (discontinued treatment to facilitate wound healing). For 3 treatments no RECIST evaluation was available to assess progression of disease and clinical progression as assessed by the treating physician was used instead.

The only factor indicating a poor PFS in the exploratory univariate analysis was the type of prior radiotherapy used: hadron radiotherapy use (carbon ion or proton beam) was associated with a worse prognosis compared to non‐hadron radiotherapy (HR = 0.39, 95% CI 0.21–0.73, *p*‐value 0.003); see Table 4.

**TABLE 4:** Prognostic factors for progression free survival (exploratory analysis).

Variables		Median PFS (months)	Univariate analysis	
		[95% CI]	Hazard ratio [95% CI]	*p*
Age (years)	< 55	5.0 [4.0–9.0]	0.73 [0.46–1.15]	0.172
	≥ 55	8.5 [5.0–12.0]	Reference	
	< 60	5.0 [4.0–9.0]	0.64 [0.39–1.04]	0.071
	≥ 60	10.0 [6.0–16.0]	Reference	
Sex	Male	8.0 [5.0–12.0]	Reference	0.164
	Female	5.5 [4.0–10.0]	1.40 [0.87–2.23]	
Histological subtype	Conventional	7.0 [5.0–10.0]	Reference	0.153
	Non‐conventional (*N* = 2)	56.0 [3.0–109.0]	0.23 [0.03–1.72]	
Primary location	Sacral	7.5 [5.0–14.0]	Reference	0.148
	Non‐sacral	5.0 [4.0–9.0]	0.71 [0.45–1.13]	
	Skull base	4.5 [2.0–10.0]	Reference	0.117
	Non‐skull base	7.0 [5.0–10.0]	1.65 [0.88–3.09]	
ECOG performance status	0–1	7.0 [5.0–10.0]	1.13 [0.54–2.37]	0.753
	2	5.5 [2.0–31.0]	Reference	
Interval since diagnosis	< 48 months	7.0 [5.0–14.0]	1.27 [0.79–2.04]	0.319
	≥ 48 months	6.0 [4.0–10.0]	Reference	
	< 60 months	7.0 [4.0–10.0]	0.95 [0.60–1.50]	0.824
	≥ 60 months	6.5 [4.0–11.0]	Reference	
Local control	Yes	10.0 [4.0–20.0]	Reference	0.260
	No	6.0 [4.0–10.0]	0.72 [0.41–1.28]	
Metastases	Yes	7.5 [4.0–11.0]	1.14 (0.72–1.81]	0.569
	No	6.0 [4.0–10.0]	Reference	
Lung metastases	Yes	7.0 [5.0–13.0]	1.10 [0.66–1.83]	0.719
	No	6.5 [4.0–10.0]	Reference	
Liver metastases	Yes	10.0 [2.0–18.0]	1.08 [0.53–2.17]	0.837
	No	6.0 [5.0–9.0]	Reference	
Bone metastases	Yes	7.5 [4.0–13.0]	0.92 [0.53–1.57]	0.746
	No	6.0 [5.0–10.0]	Reference	
Lymph node metastases	Yes	4.0 [1.0–33.0]	1.70 [0.80–3.62]	0.142
	No	7.0 [5.0–10.0]	Reference	
Subcutaneous metastases	Yes	7.5 [2.0–26.0]	1.07 [0.51–2.24]	0.864
	No	7.0 [5.0–10.0]	Reference	
Muscle metastases	Yes	6.0 [4.0–31.0]	1.58 [0.80–3.10]	0.185
	No	7.0 [5.0–10.0]	Reference	
Prior surgery	Yes	7.0 [5.0–10.0]	1.62 [0.65–4.01]	0.301
	No	5.0 [2.0–15.0]	Reference	
Resection status	R0 resection	7.5 [4.0–29.0]	1.23 [0.65–2.35]	0.528
	No R0 resection	6.0 [4.0–10.0]	Reference	
Prior radiotherapy	Yes	7.0 [5.0–10.0]	1.09 [0.54–2.19]	0.816
	No	5.0 [1.0–15.0]	Reference	
Type radiotherapy	Hadron therapy	4.0 [2.0–9.0]	0.41 [0.22–0.74]	**0.003**
	No hadron therapy	7.0 [5.0–13.0]	Reference	
Prior chemotherapy	Yes	10.0 [2.0–31.0]	1.18 [0.54–2.57]	0.685
	No	6.0 [4.0–10.0]	Reference	

*Note:* Bold indicates significant values (*p* < 0.05).

Abbreviations: CI: confidence interval, ECOG: eastern cooperative oncology group, PFS: progression free survival.

### 3.4 Overall Survival

**FIGURE 3:**
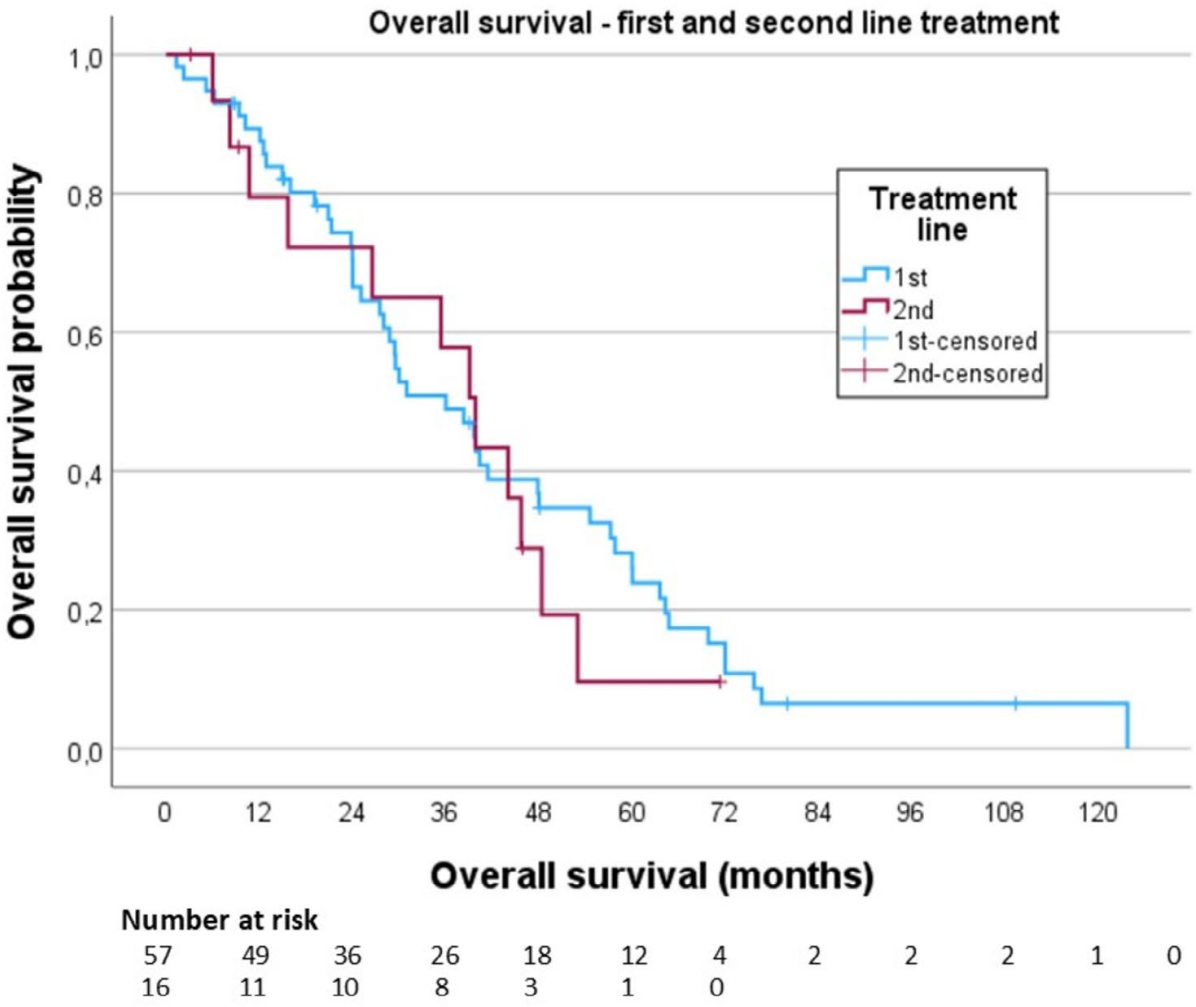
Overall survival for first and second line treatment. Median overall survival: 1st line 29.5 months (95% CI 24.0–40.4); 2nd line 37.2 months (95% CI 9.3–45.9). CI: confidence interval; PFS: progression free survival.

Median OS in first‐line treatment was 29.5 months (95% CI 24.0–40.4). In second line this was 37.2 months (95% CI 9.4–45.9), in third line 20.8 months (95% CI 12.0–35.0) and the patient treated in fourth line showed an OS of 8.0 months; see Table 3 and Figure 3.

Median OS for imatinib treatment was 30.0 months (95% CI 24.0–40.4), imatinib + sirolimus 39.1 months (95% CI 10.2–71.3), pazopanib 29.5 months (95% CI 15.7–69.8), sunitinib 16.5 months (95% CI 12.0–48.4), sorafenib 7.2 months (95% CI 3.2–39.8) and afatinib 9.4 months (95% CI 6.0–53.0); see Table 3.

Factors indicating a poorer prognosis in the exploratory univariate analysis were skull‐base primary localization of the chordoma (HR = 2.24, 95% CI 1.09–4.64, *p*‐value 0.036) and worse ECOG performance status (HR = 3.00, 95% CI 1.32–6.79, *p*‐value 0.003); see Table 5.

**TABLE 5:** Prognostic factors for overall survival (exploratory analysis).

Variables		Median OS (months)	Hazard ratio [95% CI]	*p*
		[95% CI]		
Age (years)	< 55	25.8 [15.0–39.7]	0.85 [0.52–1.38]	0.505
	≥ 55	35.4 [24.0–45.7]	Reference	
	< 60	28.8 [16.5–39.7]	0.77 [0.47–1.33]	0.371
	≥ 60	33.5 [22.4–47.9]	Reference	
Sex	Male	31.0 [25.1–41.4]	Reference	0.664
	Female	23.1 [12.1–39.9]	1.12 [0.67–1.87]	
Histological subtype	Conventional	29.5 [23.8–39.7]	Reference	0.082
	Non‐conventional	99.7 [75.7–123.8]	0.17 [0.02–1.25]	
Primary location	Sacral	38.7 (28.8–44.7]	Reference	0.105
	Non‐sacral	22.4 [12.6–30.0]	0.66 [0.40–1.09]	
	Skull base	11.4 [8.4–23.8]	Reference	**0.036**
	Non‐skull base	35.4 [27.5–40.4]	2.18 [1.05–4.50]	
ECOG performance status	0–1	29.1 [23.8–39.9]	3.18 [1.46–6.90]	**0.003**
	2	15.4 [2.3–40.4]	Reference	
Interval since diagnosis	< 48 months	39.7 [24.0–60.0]	1.37 [0.82–2.27]	0.231
	≥ 48 months	24.6 [16.0–38.3]	Reference	
	< 60 months	29.5 [19.5–41.4]	1.04 [0.64–1.71]	0.871
	≥ 60 months	27.5 [20.8–40.4]	Reference	
Local control	Yes	40.0 [15.0–48.1]	Reference	0.772
	No	28.0 [20.9–39.1]	0.91 [0.48–1.72]	
Metastases	Yes	39.4 [26.5–48.1]	1.26 [0.77–2.08]	0.356
	No	24.0 [15.0–35.4]	Reference	
Lung metastases	Yes	39.1 [21.3–45.9]	0.90 [0.52–1.54]	0.691
	No	26.3 [19.5–38.3]	Reference	
Liver metastases	Yes	31.0 [15.7–64.3]	0.80 [0.39–1.62]	0.527
	No	29.1 [21.3–39.7]	Reference	
Bone metastases	Yes	39.1 [16.5–53.0]	0.85 [0.49–1.48]	0.574
	No	28.2 [20.8–38.3]	Reference	
Lymph node metastases	Yes	46.9 [8.0–75.7]	1.58 [0.75–3.34]	0.231
	No	28.0 [21.3–38.3]	Reference	
Subcutaneous metastases	Yes	21.8 [12.9–64.7]	0.80 [0.36–1.76]	0.579
	No	29.5 [24.0–39.7]	Reference	
Muscle metastases	Yes	44.0 [12.0–72.0]	0.99 [0.52–1.90]	0.976
	No	28.4 [21.3–39.1]	Reference	
Prior surgery	Yes	30.5 [24.0–39.8]	1.19 [0.28–4.99]	0.811
	No	15.2 [9.4–29.5]	Reference	
Resection status	R0 resection	39.9 [24.0–60.0]	1.33 [0.66–2.68]	0.472
	No R0 resection	24.6 [19.5–30.3]	Reference	
Prior radiotherapy	Yes	29.7 [23.8–39.8]	1.24 [0.61–2.52]	0.548
	No	25.1 [9.4–40.8]	Reference	
Type radiotherapy	Hadron therapy	14.0 [9.4–39.8]	0.67 [0.32–1.39]	0.277
	No hadron therapy	35.7 [26.5–45.7]	Reference	
Prior chemotherapy	Yes	24.0 [8.2–39.9]	0.44 [0.18–1.05]	0.064
	No	29.8 [23.8–39.8]	Reference	

*Note:* Toxicity and dose adaptions. Bold indicates significant values (*p* < 0.05).

Abbreviations: CI: confidence interval, ECOG: eastern cooperative oncology group, PFS: progression free survival.

The two patients with dedifferentiated chordoma were treated with imatinib in first line and observed PFS was 3 and 109 months respectively.

Dose reductions were effectuated in 15/79 (19.0%) treatments. Dose interruptions occurred in 22/79 (27.8%) patients; see Table 6. Adverse events were not available in the majority of registrations and are therefore not reported.

**TABLE 6:** Dosing adaptions per MTT.

Agent	Dose reduction, *n* (%)			Dose interruption, *n* (%)			
	Yes	No	Unknown	Yes	No	Unknown	Total, *n*
Imatinib	5 (10.2)	36 (73.5)	8 (16.3)	11 (22.2)	24 (49.0)	14 (28.6)	49
Imatinib + sirolimus	3 (27.3)	8 (72.3)	—	5 (45.5)	5 (45.5)	1 (9.1)	11
Pazopanib	4 (57.1)	3 (42.9)	—	2 (28.6)	5 (71.4)	—	7
Sunitinib	2 (66.7)	1 (33.3)	—	—	1 (33.3)	2 (66.7)	3
Sirolimus + metformin	—	1 (100.0)	—	1 (100.0)	—	—	1
Sorafenib	—	4 (100.0)	—	1 (25.0)	2 (50.0)	1 (25.0)	4
Erlotinib	—	1 (100.0)	—	—	—	1 (100.0)	1
Afatinib	1 (33.3)	2 (66.7)	—	2 (66.7)	1 (33.3)	—	3
Total	15 (19.0)	56 (70.9)	8 (10.1)	22 (27.8)	38 (48.1)	19 (24.1)	79

Reasons for treatment discontinuation were progression of disease in the majority of treatments (74.7% showed radiological disease progression, 3/79, 3.8% clinical progression); see Table 7.

**TABLE 7:** Reasons for treatment discontinuation and dose adaptions.

Characteristics per individual treatment line		*n* = 79 (%)
Reason for treatment continuation	Progressive disease (radiological)	59 (74.7)
	Progressive disease (clinical)	3 (3.8)
	Toxicity	4 (5.1)
	Wound healing	3 (3.8)
	Neo‐adjuvant treatment	2 (2.5)
	Patient decision	1 (1.3)
	Died another reason than PD	1 (1.3)
	Reason unknown	5 (6.3)
	Ongoing on medication	1 (1.3)
Reason dose reduction/interruption (most frequently reported)	Measured blood level of drug	4 (5.1)
	Gastro‐intestinal toxicity	3 (3.8)
	Hepatic toxicity	2 (2.5)
	Renal toxicity	2 (2.5)
	Skin toxicity	1 (1.3)
	Other	15 (19.0)

## 4 Discussion

In this retrospective series, 57 AC patients were treated with seven different MTTs. Patients received treatment in first (*n* = 57), second (*n* = 16), third (*n* = 5) or fourth (*n* = 1) line. Most frequently administered agent in first line was imatinib (84.2%) and in second line imatinib + sirolimus (35.7%). Overall median PFS and OS in first‐line treatment was 6.5 (95% CI 4.0–10.0) and 29.5 months (95% CI 24.0–40.4) and in second line 10.0 (95% CI 4.0–22.0) and 37.2 months (95% CI 9.4–45.9). A partial response according to RECIST was seen in 5/79 treatments (6.3%). Pazopanib and imatinib in combination with sirolimus proved the most effective treatments in our series.

The longer PFS and OS in the second‐line treatment group are surprising and could be attributed to the efficacy of the imatinib + sirolimus combination. However, since multiple treatments were delivered in fewer individual patients, this is more likely attributable to a selection of fitter subjects with a more favourable tumour biology that were eligible for second line treatment. The association between hadron radiotherapy and reduced progression‐free survival could be linked to the predominance of primary tumours in the skull base or cervical spine among these patients, since these patients are more often primarily treated with radiotherapy and are known to have a higher relapse rate after primary treatment [11]. Due to the small sample size, the prognostic factor analysis presented should be interpreted as exploratory only. The observed associations of treatment‐related factors and outcomes are vulnerable to confounding and case selection.

Previous large case series reporting on MTT in chordoma patients have described treatment outcomes of imatinib comparable to our series results [12, 13, 14, 15, 16]. In the largest retrospective series by Lebellac et al. 80 patients were reported of whom 62 received imatinib [13]. A partial RECIST response was seen in 5/80 (6.3%) of subjects, of whom 3 received imatinib. Median PFS and OS were longer with 9.4 months and 4.4 years respectively. In another series by Hindi et al. no RECIST responses were seen in 48 patients on imatinib and median PFS was 9.9 months [12]. In the largest phase 2 trial on imatinib in 50 AC patients by Stacchiotti et al., a median PFS of 9 months, an objective response rate of 2% and clinical benefit in 64% of patients was seen. In 10/26 (39%) of subjects evaluated by PET‐CT a maximum standard uptake value decrease ≥ 25% was observed at 3 months [9]. Further small phase II trials with sorafenib (multikinase inhibitor; PDGFRβ, VEGFR2‐3, RAF family), lapatinib (EGFR and HER2 inhibitor) and afatinib [17] have not been able to show a more favourable outcome [18, 19]. The vascular endothelial growth factor receptor‐2 (VEGFR‐2) inhibitor apatinib was able to reach a longer median PFS of 18 months in a small phase II trial; nonetheless the objective response rate did not outperform other MTTs [20].

A range of other MTTs have been described in previous case reports and small case series, like sunitinib [13, 21, 22], dasatinib [23], combination of imatinib and sirolimus [15], pazopanib [22, 24], erlotinib [13, 25, 26, 27, 28, 29] and regorafenib [30]. So far, no clinically relevant biomarkers have been identified for the treatment of AC More recently treatment with immunotherapy and vaccines (e.g., brachyury) has been studied with varying results [31, 32, 33, 34]. Tables 8 and 9 provide an overview of previously published studies, including MTT trials, retrospective series and case reports.

**TABLE 8:** Overview of phase I/II studies on MTT in chordoma.

Author [References]	Drug	*N* ^a^	Study type	Arm	Year	Journal	Result	Comments
Imatinib								
Heinrich et al. [35]	Imatinib	5	Phase II	1	2008	Clin Cancer Res	Objective response: SD 4/5	All solid tumours
							Median PFS—(range 2.7–33.0)	
Stacchiotti et al. [9]	Imatinib	50	Phase II	1	2012	J of Clin Oncology	Objective response: PR 2%, 70% SD, 64% clinical benefit	
							Median PFS 9.0 months	
Adenis et al. [36]	Imatinib and cyclophosphamide	7	Phase I	1	2013	British J Cancer	Median PFS 10.2 months (range 4.2–18)	All solid tumours
Stacchiotti et al. [37]	Imatinib and everolimus	43	Phase II	1	2018	Cancer	ORR 9/43 (20.9%)	
							Median PFS by Choi 11.5 months	
							Median PFS RECIST 14 months	
							Median OS RECIST 47.1 months	
Other TKI (target)								
Stacchiotti et al. [19]	Lapatinib (EGFR/Her2)	18	Phase II	1	2013	Ann Oncology	Response rate: – RECIST: 83.3% SD, 16.7% PD – Choi: 4/10 response, 1/10 SD, 5/10 PD	
							Median PFS: – RECIST: 8 months (range 4–12) – Choi: 6 months (range 3–8)	
Bompas et al. [18]	Sorafenib (multi: RAS, VEGF a.o.)	27	Phase II	1	2015	Ann Oncology	Response rate: – RECIST: 1/27 PR, 24/27 SD, 1/27 PD, 1 not assessable – Choi: 7/27 PR, 5/27 SD, 1/27 PD, 14 not assessable	
George et al. [21]	Sunitinib (multi: PDGFR, VEGF a.o.)	9	Phase II, prospective	1	2009	J Clin Oncology	RECIST response: – SD at 16 weeks 4/9 – SD at 24 weeks 2/9	Non‐GIST soft tissue sarcoma
Schuetze et al. [23]	Dasatinib (multi: BCR‐ABL, PDGFR a.o.)	20	Phase II	1	2017	Cancer	6‐month PFS 54% Choi objective response 6/20	ASPS, CS, chordoma, epithelioid sarcoma, SFT
Chi et al. [38]	Tazemetostat (EZH2)	4	Phase I	1	2018	JCO (ASCO abstract)	Objective response in 2/4	INI1‐negative tumours in paediatric population
Cote et al. [39]	Nilotinib and radiotherapy (multi: BCR‐ABL, PDGFR a.o.)	23	Phase I	1	2018	Int J Radiation Onc Biol Physics	Median PFS 58.15 months Median OS 61.5 months 2‐year OS 95%	High‐risk non metastatic chordoma
Liu et al. [20]	Apatinib (VEGFR2)	30	Phase II	1	2020	Lancet Oncology	ORR RECIST 1/27 (3.7%) ORR Choi 6/27 (25.9%) Median PFS RECIST/Choi 18 months	
Le Cesne et al. [30]	Regorafenib (multi: VEGF, BRAF, PDGFR a.o.)	27	Phase II Randomised	2	2023	ESMO Open	Median PFS 8.2 months regorafenib vs. 10.1 months placebo Median OS 28.3 months regorafenib vs. not reached placebo group	
Lipplaa et al. [17]	Afatinib (HER2)	47	Phase II	1	2024	JCO (ASCO abstract)	Median PFS 8.6 months ORR RECIST 4/43 (9.3%)	
Gounder et al. [40]	ERAS‐601 and cetuximab (SHP2 and EGFR)	11	Phase I	2	2024	JCO (ASCO abstract)	ORR RECIST 1/9 (11.1%) in ERAS‐601 + cetuximab group	

Abbreviations: a.o.: among others, ASPS: alveolar soft part sarcoma, CS: chondrosarcoma, EGFR: epidermal growth factor receptor, mTOR: mammalian target of rapamycin, ORR: overall response rate, OS: overall survival, PDGFR: platelet derived growth factor‐receptor, PD: progressive disease, PFS: progression free survival, PR: partial response, RECIST: response evaluation criteria in solid tumours, SD: stable disease, SFT: solitary fibrous tumour, TKI: tyrosine kinase inhibitor, VEGFR: vascular endothelial growth factor receptor.

^a^ Number of treated subjects with a chordoma diagnosis.

**TABLE 9:** Overview of retrospective case series and reports MTT in chordoma.

Author [References]	Drug	*N* ^a^ ^s^	Study type	Date	Journal	Result
Imatinib						
Lebellec et al. [13]	Imatinib	62	Case series, retrospective	2017	European J Cancer	Objective response: PR 3/62, SD 43%
						Median PFS 9.4 months (95% CI 6.8–16.1)
	Sorafenib (multi: RAS, VEGF, FLT3 a.o.)	11				Objective response: PR 1/11, SD 9/11
	Sunitinib (multi: VEGF, PDGFR a.o.)	1				Objective response: SD 1/1
	Temsirolimus (mTOR)	1				Objective response: SD 1/1
	Erlotinib (EGFR)	5				Objective response: PR 1/5, SD 4/5
Hindi et al. [12]	Imatinib	48	Case series, retrospective	2015	European J Cancer	Objective response: SD 74%, PD 26%
						Median PFS: 9.9 months (95% CI 6.7–13)
Casali et al. [14]	Imatinib	6	Case series, retrospective	2004	Cancer	Symptomatic improvement 4/5 symptomatic pts. PET response 1/6
Stacchiotti et al. [15]	Imatinib + sirolimus	10	Case series, retrospective	2009	Ann Oncology	Response at 3 months: – RECIST 1/9 pts. PR, 7/9 pts. SD, 1/9 pts. PD – PET/Choi response 7/9 pts. – Clinical benefit rate 89%
Baldi et al. [16]	Weekly cisplatin ± imatinib	33	Case series, retrospective	2022	Cancer	Median OS 30.3 months
						Median PFS 8.0 months
Other TKI (target)						
Shinji et al. [24]	Pazopanib (multi: VEGF, KIT, PDGFR a.o.)	1	Case report	2016	Gan To Kagaku Ryoho	PFS 14 months, clinical benefit
Lipplaa et al. [22]	Pazopanib	4	Case series, retrospective	2016	Clinical Sarcoma Research	Pazopanib: RECIST response: 2/4 pts. SD at 14 and 15 months. Median PFS 8.5 months.
	Sunitinib (multi: PDGFR, VEGF, cKIT a.o.)	1				Sunitinib: RECIST response: 1/1 patients PR, PFS 27 months.
Ricci‐Vitiani et al. [41]	Sirolimus (mTOR)	1	Case report	2013	Neoplasia	PD, tumour growth rate decreased
Trapani et al. [29]	Erlotinib	1	Case report	2017	Translational Medicine	PFS 8 weeks
Houessinon et al. [28]	Erlotinib	1	Case report	2015	Case Rep Oncology	Partial response RECIST
						PFS 28 months
Launay et al. [27]	Erlotinib	1	Case report	2011	BMC Cancer	PFS 12 months
Singhal et al. [42]	Erlotinib	1	Case report	2009	Anti‐cancer Drugs	Partial response RECIST
						PFS 11 months
Asklund et al. [25]	Erlotinib + bevacizumab (VEGF)	3	Case series, retrospective	2014	Acta Oncologica	PFS 27–51 months
Aleksic et al. [26]	Erlotinib + linsitinib (IGF‐1R)	1	Case report	2016	Frontiers Oncology	PFS 60 months
Hof et al. [43]	Cetuximab (EGFR) + gefitinib (EGFR)	1	Case report	2006	Onkologie	Partial response RECIST
						PFS 9 months
Lindén et al. [44]	Cetuximab + gefitinib	1	Case report	2009	Acta Oncologica	Clinical improvement and tumour regression after 4 months
Liang et al. [45]	Crizotinib (ALK)	1	Conference paper	2015	ASCO annual congress	PFS 17 months
Mir et al. [46]	Erlotinib	31	Conference Poster	2021	ASCO annual congress	ORR 4/31 (13%)
						Median PFS 6.2 months
						Median OS 15.9 months

Abbreviations: ALK: anaplastic lymphoma kinase, a.o.: among others, EGFR: epidermal growth factor receptor, IGF‐1R: insulin‐like growth factor‐1 receptor, mTOR: mammalian target of rapamycin, ORR: overall response rate; OS: overall survival, PD: progressive disease, PDGFR: platelet derived growth factor‐receptor, PFS: progression free survival, PR: partial response, RECIST: response evaluation criteria in solid tumours, SD: stable disease, TKI: tyrosine kinase inhibitor, VEGFR: vascular endothelial growth factor receptor.

^a^ Number of treated subjects with a chordoma diagnosis.

The main limitation of this study is its small sample size and retrospective nature. However, given the challenges of setting up trials in an ultra‐rare disease such as chordoma, these larger retrospective series provide very useful insights for treating oncologists and adds to the limited prospective research data. In addition to the very small patient numbers, the use of different MTT's, treatment centre and physician's personal preferences have led to heterogenous second‐ and third‐line treatments that make it difficult to directly compare treatments head‐to‐head. Furthermore, symptomatic improvement was not consistently reported and no standardised measurement tool of questionnaire was used. Treatment outcomes were defined solely by RECIST responses and (progression free) survival duration. In a typically slowly‐progression neoplasm such as chordoma RECIST response might not be ideal in assessing tumour responses [47, 48]. Consequently an increasing number of investigators use Choi response criteria to evaluate response in chordoma, also taking into account tumour density changes, instead of volumetric assessment alone [19, 20, 23, 37]. A recent meta‐analysis by Meng et al., evaluating the efficacy and safety of 7 different MTTs in chordoma reported an objective response rate (ORR) of 1.7% measured by RECIST 1.1 [49]. When using Choi criteria, a much higher response rate of 27% was observed. In addition, adapted Choi criteria have proven to be better at predicting pathologic tumour response compared to RECIST criteria in a pilot‐study by Stacchiotti in advanced soft tissue sarcoma [50]. The role of [^18^F]‐fluorodeoxyglucose PET scans in chordoma is yet to be defined, but seems promising in determining metabolic response from the limited evidence available [4, 51, 52]. Given the slow growing rate of chordoma, ideally more prospective studies would include a randomised design to a placebo arm (with crossover), which would provide a valuable comparison and enable a more informed discussion with patients about the relative benefits/risks of treatment versus no treatment.

Dose reductions and dose interruptions were seen in 19% and 26.6% of patients respectively and 5.1% of patients discontinued MTT due to toxicity, which is in line with dose adaptations reported in previous MTT trials [49]. Treatment tolerability and differences between the MTTs were not evaluable given the small number of non‐imatinib treated subjects and missing adverse events registration in this series.

Immunotherapy is a relatively new player in the field of chordoma. The first phase 2 trial by Blay et al. tested PD1 inhibitor pembrolizumab in 34 chordoma patients and showed a partial response in 4/34 (12%) patients and median PFS of 6.1 months [53]. A trial by DeMaria combined PD(L)‐1 inhibitors with tremelimumab (CTLA‐4 inhibitor) and relatlimab (LAG3 inhibitor) respectively, leading to significantly longer median PFS of 13.6 months [33]. The toxicity was in line with what has been previously reported for anti‐CTLA‐4 and anti‐PD‐1 agents.

Three vaccine studies have been published so far, first of all a phase 1 trial by Heery et al. testing a brachyury vaccine (GI‐6301) in 11 chordoma patients. A median PFS of 8.3 months and partial response in 1/10 evaluable patients was seen [54]. DeMaria and colleagues further tested the GI‐6301 vaccine in a phase 2 trial, in which 24 subjects were randomised double‐blind between vaccine or placebo followed by radiotherapy hoping to achieve a better immune response [31]. Tables 10 and 11 provide an overview of immunotherapy trials, retrospective series and case reports.

**TABLE 10:** Overview of phase I/II studies in immunotherapy for chordoma.

Author [References]	Drug	Target	*N* ^a^	Study type	Arms	Year	Journal	Result	Comments
Heery et al. [54]	Brachyury vaccine (GI‐6301)	Brachyury	11	Phase I	1	2015	Cancer Immunolog Research	Median PFS 8.3 months	All solid tumours
								RECIST response: 1/10 PR	
Blay et al. [53]	Pembrolizumab	PD‐1 receptor	34	Phase II	1	2021	Lancet Oncology	RECIST response (PR): 4/34 (11.8%)	Ultra rare sarcoma
								Median PFS 6.1 months	
								1‐year OS 76.6%	
DeMaria et al. [31]	Brachyury vaccine + radiotherapy	Brachyury	24	Phase II (randomised, double‐blind)	2	2021	Oncologist	RECIST response: 1 PR in both arms	
								Median PFS: 20.6 months vaccine arm, 25.9 months placebo arm, HR 1.02.	
Somaiah et al. [33]	Durvalumab and tremeli‐ mumab	PDL‐1 receptor, CTLA‐4	5	Phase II	1	2022	Lancet Oncology	Median PFS 13.57 months	Advanced and metastatic sarcoma
								12‐week PFS rate 0.8	
Cote et al. [34]	Brachyury vaccine + radiotherapy	Brachyury	26	Phase II	1	2024	Cancer	RECIST response: PR in 2/26, SD 21/26.	
								Median PFS not reached during study.	
Burkenroad et al. [55]	Nivolumab + relatlimab	PD‐1, LAG‐3	10	Signal finding	1	2024	ASCO conference	Median PFS 4.9 months	Closed prematurely due to slow accrual
								RECIST response: 1/9	
								PFS rate at 6 months: 44.4%	

Abbreviations: CTLA‐4: cytotoxic lymphocyte associated protein 4, HR: hazard ratio, LAG3: lymphocyte‐activation gene 3, PD(L)‐1: programmed cell death (ligand) protein 1, PFS: progression free survival, PR: partial response, RECIST: response evaluation criteria in solid tumours, SD: stable disease.

^a^ Number of treated subjects with a chordoma diagnosis.

**TABLE 11:** Overview of retrospective case series and reports on immunotherapy in chordoma.

Author [References]	Drug	Target	*N*	Study type	Date	Journal	Result
Jagersberg et al. [56]	Nivolumab + pazopanib	PD‐1 + VEGFR, PDGFR	1	Case report	2017	Acta Neurochirurgica	PFS 2 years
Migliorini et al. [57]	1. Pembrolizumab	PD‐1	3	Case series	2017	Oncoimmunology	1. PFS 6 months
	2. MVX‐ONCO‐1 (personalised immunotherapy)						2. PR, PFS 19 months
	3. Nivolumab						3. PFS 9 months
Wu et al. [58]	Pembrolizumab	PD‐1	1	Case report	2020	Frontiers Oncology	PFS 9.3 months
Bishop et al. [59]	Pembrolizumab, durvalumab + tremelimumab, nivolumab + IL2, FAZ053	PD‐1, PD‐L1, CTLA‐4	17	Case series	2022	Immunotherapy Journal	RECIST response: CR 6%, PR 18%, SD 65%
							Median PFS 14 months
Kesari et al. [60]	AdAPT‐001 + nivolumab	PD‐1	1	Case report	2023	Case Reports in Oncology	PFS 7 months

Abbreviations: CTLA‐4: cytotoxic lymphocyte associated protein 4, IL2: interleukine 2, PDGFR: platelet derived growth factor‐receptor, PD(L)‐1: programmed cell death (ligand) protein 1, PFS: progression free survival, PR: partial response, RECIST: response evaluation criteria in solid tumours, SD: stable disease, VEGFR: vascular endothelial growth factor receptor.

Results from several ongoing clinical trials conducted by other investigators evaluating MTTs are currently awaited, such as the anti‐EGFR agent cetuximab (https://clinicaltrials.gov/study/NCT05041127), CDK4/6 inhibitor palbociclib (https://clinicaltrials.gov/study/NCT03110744) and a trial comparing anlotinib (anti‐VEGFR) and imatinib head to head in AC (https://clinicaltrials.gov/study/NCT04042597). Combination treatments like pemetrexed plus pembrolizumab (https://clinicaltrials.gov/study/NCT06794645) and apatinib (anti‐VEGFR) plus camrelizumab (anti PD‐1 https://clinicaltrials.gov/study/NCT06140732) are currently under investigation as well.

## 5 Conclusion

This retrospective series describes 57 patients treated with a single or multiple lines of MTT. Results are in line with previous phase 2 trials and retrospective series showing comparable PFS results and few objective responses according to classic RECIST criteria. Given their limited response rate and considerable toxicity, most of these agents do not meet the ESMO Magnitude of Clinical Benefit Scale (MCBS) criteria for single arm studies in orphan diseases or diseases with a high unmet need assessing the clinical benefit of new cancer therapies [61, 62]. However MTTs are frequently used off‐label due to the lack of other systemic treatment options. The toxicity profile and limited efficacy rate should be taken into account in counselling patients. Further research is needed to explore the efficacy of other targeted therapies and immunotherapy or a combination of both treatment modalities.

## Author Contributions

**Ioannis Boukonavis:** investigation, writing – review and editing. **Javier Martinez‐Trufero:** investigation, writing – review and editing. **Josefina Cruz:** investigation, writing – review and editing. **Wilco Peul:** investigation, writing – review and editing. **Nadia Hindi:** investigation, writing – review and editing. **Pawel Teterycz:** investigation, writing – review and editing. **Robin J. Young:** investigation, writing – review and editing. **Robert J. P. van der Wal:** investigation, writing – review and editing. **Frank Speetjens:** investigation, writing – review and editing. **Hans Gelderblom:** conceptualization, investigation, writing – review and editing, supervision. **Astrid Lipplaa:** conceptualization, investigation, writing – original draft, methodology, writing – review and editing, formal analysis, project administration, data curation.

## Funding

The authors have nothing to report.

## Ethics Statement

The study was approved by the local Ethics Committee at the Leiden University Medical Center in Leiden, The Netherlands. Written consent was not required from patients in compliance with Dutch laws governing non‐interventional studies.

## Consent

The authors have nothing to report.

## Conflicts of Interest

The authors declare no conflicts of interest.

## Data Availability

The data that support the findings of this study are available from the corresponding author upon reasonable request.
